# Supplementary material for: Chromothripsis during telomere crisis is independent of NHEJ, and consistent with a replicative origin
Source: Genome Res. 2019 May;29(5):737–49. doi: 10.1101/gr.240705.118 (PMC6499312; doi:10.1101/gr.240705.118)
Supplement: Supplemental Material [file supp_gr.240705.118_Supplemental_file_1.zip › contigs/annotated_contigs/DB39/contig.2.DB39_length_624_mean_cov_4.94391025641.docx]

**DB39_length_624_mean_cov_4.94391025641**

```
AAACCACACTTATCCTCAAACTTCACAGTAAGCCCACAGCAGCCCTGCCCAGGGAGAGGGCCTGTCAGCCGGGTACCCTGTGTCCAGGG
 >chr17:27082701-27082919 + E=2e-119
TTTCATTGAAGAACTGTCTACAATCACCATAGCACCTGGCCTTAAATAATGGATTTGGAGACGGCTTCTGGGTGGGATGCATATTACAG

GGGTACCCCTTTTAAAAATTAGGAATTACTCCTATGAAGT|AAGGCATGACTTGCAGCTGTCTCCTGGATTGTGTCATCATGGGGTATC
                                          >chr17:27069077-27069394 + E=5e-179
AGGGCTGGCAAAACCCCTGCTCTGCTTTTGGCCTTGGATATGGAAACCTCAACCACTTTGTTCTCCTACCACCTCTTTGCCCTTCCTAC

CCCTTCCTCCCTTCAGTCAGTGTCCCCAGGGTCCAGCCTGGCTAGAGTTAGAAGGCAGACCTAGCCTTTGGAAGCAGGGTGGCCTCCAG

AGCCTTGCCATAAAAAGGCTGAAGGCAGCCGGGCGCAGTGGCTCACGCCTGTAATCCCAGCACTTTGGGAGGCTGAGGCGGGCGGATCA

CG|TGAGCTCAGTAGACTGAGGCGAGCGGGGCCAACGGGGTGAAACAGATCGGAAGAGCGGCGTGGAGGGAAAGGGTGGGTGAGGACGT

GGA
```
